# Relationship between sarcopenia and cardiovascular disease risk among Taiwanese older adults

**DOI:** 10.1017/S1368980022000684

**Published:** 2022-07

**Authors:** Yuan-Yuei Chen, Wei-Liang Chen, Tao-Chun Peng, Fang-Yih Liaw, Yuan-Ping Chao, Tung-Wei Kao

**Affiliations:** 1 Department of Pathology, Tri-Service General Hospital and School of Medicine, National Defense Medical Center, Taipei, Taiwan, Republic of China; 2 Department of Pathology, Tri-Service General Hospital Songshan Branch and School of Medicine, National Defense Medical Center, Taipei, Taiwan, Republic of China; 3 Division of Family Medicine, Department of Family and Community Medicine, Tri-Service General Hospital and School of Medicine, National Defense Medical Center, Taipei, Taiwan, Republic of China; 4 Division of Geriatric Medicine, Department of Family and Community Medicine, Tri-Service General Hospital and School of Medicine, National Defense Medical Center, 114 Taipei, Taiwan, Republic of China; 5 Department of Biochemistry, National Defense Medical Center, Taipei Taiwan, Republic of China

**Keywords:** Sarcopenia, dynapenia, Framingham risk score

## Abstract

**Objective:**

Increasing evidence supports sarcopenia as an important parameter for predicting cardiometabolic risks. The objective of this study was to investigate the relationship between muscle mass, muscle strength, and physical performance, and cardiovascular risk among older community-dwelling adults.

**Design:**

The associations between dynapenia, sarcopenia, and Framingham risk score (FRS) were estimated by multivariate regression models.

**Setting:**

Muscle mass is estimated by skeletal muscle mass index using a bioelectrical impedance analysis. Muscle strength is measured by handgrip strength using an analogue isometric dynamometer. Physical performance is measured by gait speed using a 6-m walking distance. Dynapenia was defined as low muscle strength and/or slow gait speed presents with normal muscle mass. The diagnosis of presarcopenia and sarcopenia was based on criteria proposed by the Asian Working Group for Sarcopenia in 2014. The FRS was used for evaluating 10-year coronary heart disease (CHD) risk.

**Participants:**

Adults aged 65 years and older who attended health examinations from 2015 to 2017 were recruited.

**Results:**

There were totally 709 subjects enrolled in this study. Dynapenic men (*n* 47) had 17·70 ± 5·08 % FRS and sarcopenic women (*n* 74) had 7·74 ± 6·06 % FRS. Participants with presarcopenia had the lowest FRS (men: 15·41 ± 5·35 %; women: 5·25 ± 3·70 %). Men with dynapenia had higher FRS than the presarcopenia group with odds ratio (OR) of 2·52 (95 % confidence interval (CI): 1·03, 6·14). Women with sarcopenia had significantly higher FRS than the presarcopenia group with OR of 2·81 (95 % CI: 1·09, 7·27).

**Conclusion:**

Older dynapenic men and older sarcopenic women had higher risks of 10-year CHD. Presarcopenic older adults had the lowest CHD risk in both genders.

Sarcopenia is a multifactorial condition which is characterised by the decline of muscle mass with reduced muscle strength and poor physical performance^(1)^. European Working Group on Sarcopenia in Older People (EWGSOP) demonstrated an operational definition and diagnostic strategy for sarcopenia in 2010 that had become the most widely used consensus in the world^(2)^. To reflect the severity in clinical practice, sarcopenia is suggested to be classified into conceptual staging as ‘presarcopenia’, ‘sarcopenia’ and ‘severe sarcopenia’. In Taiwan, the reported prevalence from several community studies was about 5·4–10·8 % in men and 2·5–7·8 % in women by using the EWGSOP definition^(3,4)^. Sarcopenia is associated with the risk of morbidity and disability in the elderly population, including loss of independence, poor quality of life and even death^(2,5)^. Numerous studies have reported that sarcopenia is a potential predictor of cardiometabolic health problems such as insulin resistance, dyslipidemia and CVD^(6–8)^. Dynapenia is defined as the age-related muscle strength decline that is not caused by neurologic or muscular diseases^(9)^. The consequences of dynapenia are including increased risks of physical disability, poor physical performance and even death. Several evidences have reported a direct relationship between low muscle strength, metabolic syndrome and insulin resistance^(10)^. In a cross-sectional study, dynapenia is proposed to be associated with increased risks of CVD, which may lead to incident cardiovascular events^(11)^.

CVD is the leading cause of death in the world, and the prevalence is increasing in Taiwan^(12,13)^. Numerous studies have reported the effects of muscle quality and function on CVD risk. Low muscle mass is associated with coronary calcification and considered an independent risk of coronary heart disease (CHD)^(14)^. In a prospective cohort study, muscle strength is suggested to be more important for CVD risk than muscle mass^(15)^. Furthermore, sarcopenia is reported to be associated with increased CVD risk and may be an early predictor of its susceptibility in both elderly and middle-aged adults in Korea^(16,17)^. Among several different scores in estimating CVD risk, the Framingham risk score (FRS) is a simplified and gender-specific clinical algorithm for the assessment of the 10-year CVD risk of an individual^(18)^. Numerous studies have supported that the predictive values of the FRS for CVD events were varied in asymptomatic men and women^(19)^; in addition, the cut-off values to define different degree of dynapenia and sarcopenia in men and women were different. For these reasons, we hypothesised that gender is a potential clinical factor in the relation of muscle decline and CVD risks. The purpose of our study was to investigate the gender-specific difference in the relationship between different degree of sarcopenia and CVD risk in the Taiwanese community-dwelling elderly population.

## Method

### Study population

In this cross-sectional study, we included participants who attended health check-up at Tri-Service General Hospital (TSGH) in Taiwan during the period of 2015–2017. All participants were asked to complete questionnaires, measurements of muscle mass and muscle strength, and laboratory examinations. The exclusion criteria were that participants who were younger than 65 years and missing data of self-reported questionnaires or examinations. All analytic procedures were conducted by well-trained medical staff at the health check-up centre following the guidelines of TSGH. To access data from the medical examination, participants were requested to complete written informed consent. The study design was approved by the Institutional Review Boards of TSGH, Taiwan.

### Definition and diagnosis of robust, sarcopenia, presarcopenia and dynapenia

According to the guidelines proposed by the Asian Working Group for Sarcopenia (AWGS) in 2014^(20)^, the cut-off values of skeletal muscle mass index (SMI) is 7·0 kg/m^2^ for men and 5·7 kg/m^2^ for women evaluated by using bioelectric impedance analysis (BIA, InBody720, Biospace). The definition of low handgrip strength is below 26 kg for men and lower than 18 kg for women measured by using an analogue isometric dynamometer. The indication of low gait speed is slower than 0·8 m/s which is derived from calculating the 6-m walking distance divided by time in seconds. Robust was defined as having normal SMI, normal handgrip strength and normal gait speed. Participants with normal SMI but had low handgrip strength and/or low gait speed were categorised as dynapenia. Presarcopenia was defined as low SMI with normal handgrip strength and normal gait speed; sarcopenia was indicated by low SMI with either low handgrip strength or low gait speed or both.

### Ten-year CHD Risk Scores

Several scoring systems exist to help clinicians evaluate the 10-year CHD risk score. The FRS, calculated the risk at 10 years in percent, is the most widely used globally and is applied in the present study. D’Agostino et al. outlined the Framingham algorithm to calculate the risk score based on age, gender, high-density lipoprotein cholesterol (HDL-C), total cholesterol, systolic blood pressure, and history of smoking and diabetes mellitus^(21)^. Participants were classified as never, current and former smokers by self-reported questionnaire. Diabetes mellitus was determined as self-reported medical diagnosis and/or using any hypoglycemic medication. Systolic blood pressure was estimated by a standard sphygmomanometer when the participants were seated. Serum total cholesterol and HDL-C were collected from participants after fasting for at least 8 h. The higher FRS in male and female individuals is 20 % and 10 %, respectively.

### Variable assessment

Body mass index (BMI) was estimated that the weight (kg) divided by the square of height (m) (kg/m^2^). Waist circumference was estimated in the horizontal plane between the iliac crest and the lowest rib. Body fat was measured by BIA. History of alcohol drinking, hypertension, stroke and coronary artery disease was obtained from participants’ self-reported questionnaires.

### Statistical analysis

All statistical estimations were calculated using the Statistical Package for the Social Sciences, version 22.0 (SPSS Inc.) for Windows. Continuous variables were presented as means and standard deviation (sd), while categorical variables were presented as frequencies and percentages (%). The differences between the gender groups in terms of demographic information and laboratory data were examined by ANOVA for continuous variables and chi-square test for categorical parameters. Statistical significance was defined as a two-sided *P*-value of ≤ 0·05. The association between different degrees of sarcopenia and higher FRS was analysed using logistic regression with an unadjusted and adjusted model for all pertinent variables in the study.

## Results

### Characteristics of the study population

The characteristic information of male and female population was represented in Table 1 and Table 2. A total of 305 men and 404 women were included in the final analysis of the study. The mean age of participants in robust, dynapenia, presarcopenia and sarcopenia group was 72·1 ± 6·6, 77·6 ± 8·8, 76·0 ± 8·5 and 83·9 ± 5·9 years in men and 70·2 ± 5·6, 73·6 ± 6·6, 70·9 ± 5·2 and 75·1 ± 7·1 years in women. Men with sarcopenia had significantly lower SMI, and handgrip strength, and higher body fat and fat to muscle ratio than other groups. Participants in men with dynapenia had significantly lower gait speed, total cholesterol, and higher BMI and waist circumference than other groups. In terms of women, participants with sarcopenia had significantly lower systolic blood pressure, SMI, and grip strength and higher FRS than other groups. Dynapenic female participants had significantly lower gait speed, total cholesterol and HDL-C and higher BMI, waist circumference , systolic blood pressure, SMI, body fat and fat to muscle ratio than other groups.

**Table 1:** Characteristics of male participants

	Robust (*n* 183)		Dynapenia (*n* 47)		Presarcopenia (*n* 41)		Sarcopenia (*n* 34)		Total (*n* 305)		*P*
	Mean	sd	Mean	sd	Mean	sd	Mean	sd	Mean	sd	
Continuous variables*											
Age (years)	72·1	6·6	77·6	8·8	76·0	8·5	83·9	5·9	74·8	8·1	<0·001
BMI (kg/m^2^)	25·7	2·7	26·5	3·3	22·0	2·4	22·4	2·4	25·0	3·1	<0·001
Waist circumference (cm)	86·9	9·0	88·2	10·7	78·2	8·2	81·8	8·9	85·4	9·7	<0·001
Systolic BP (mmHg)	133·5	13·7	133·4	14·2	128·5	18·8	133·4	18·1	132·8	15·1	0·280
SMI (kg/m^2^)	7·8	0·5	7·8	0·5	6·2	1·4	6·1	1·2	7·4	1·1	<0·001
Grip strength (kg)	36·5	6·6	26·2	7·0	33·2	4·7	22·1	5·2	32·9	8·2	<0·001
Gait speed (m/s)	1·35	0·3	0·92	0·3	1·30	0·2	0·93	0·2	1·23	0·3	<0·001
Body fat (%)	26·9	5·6	27·6	6·6	24·4	5·0	28·8	7·0	26·9	5·9	0·015
Fat to muscle ratio	0·68	0·1	0·73	0·2	0·61	0·1	0·80	0·2	0·69	0·2	0·001
Total cholesterol (mg/dl)	175·6	31·8	165·1	32·5	184·3	28·6	170·1	29·9	174·5	31·6	0·029
HDL-cholesterol (mg/dl)	48·5	10·5	50·8	11·5	54·9	11·4	53·5	14·8	50·3	11·5	0·003
FRS (%)	17·64	5·95	17·70	5·08	15·41	5·35	17·41	6·42	17·32	5·83	0·161
	*n*	%	*n*	%	*n*	%	*n*	%	*n*	%	
Categorical variables†											
Smoking	71	38·7	9	19·1	10	24·3	6	17·6	96	31·4	0·006
Alcohol drinking	28	15·3	4	8·5	3	7·3	1	2·9	36	11·8	0·073
Hypertension	79	43·1	30	63·8	9	21·9	14	41·1	132	43·2	0·001
Diabetes mellitus	22	12·0	10	21·2	4	9·7	5	14·7	41	13·4	0·388
Stroke	4	2·1	4	8·5	1	2·4	2	5·8	11	3·6	0·239
Metabolic syndrome	53	28·9	14	29·7	2	4·8	5	14·7	74	24·2	0·001

BP, blood pressure; BMI, body mass index; HDL, high-density lipoprotein; SMI, skeletal muscle mass index; FRS, Framingham risk scores.

* Values in the continuous variables were expressed as mean and standard deviation unless otherwise specified.

† Values in the categorical variables were expressed as number (percent).

**Table 2:** Characteristics of female participants

	Robust (*n* 191)		Dynapenia (*n* 85)		Presarcopenia (*n* 54)		Sarcopenia (*n* 74)		Total (*n* 404)		*P*
	Mean	sd	Mean	sd	Mean	sd	Mean	sd	Mean	sd	
Continuous variables*											
Age (years)	70·2	5·6	73·6	6·6	70·9	5·2	75·1	7·1	71·9	6·3	<0·001
BMI (kg/m^2^)	24·6	3·1	26·1	3·2	21·5	2·2	22·0	2·8	24·1	3·4	<0·001
Waist circumference (cm)	78·2	7·6	82·2	7·8	71·8	6·8	74·3	7·8	77·5	8·3	<0·001
Systolic BP (mmHg)	131·3	16·9	136·5	15·1	131·1	15·7	130·0	16·2	132·1	16·3	0·047
SMI (kg/m^2^)	6·38	0·4	6·41	0·6	5·32	0·3	5·06	0·9	6·01	0·8	<0·001
Grip strength (kg)	23·2	3·7	15·9	4·4	22·0	3·2	14·3	3·9	19·8	5·4	<0·001
Gait speed (m/s)	1·25	0·2	0·91	0·3	1·33	0·2	0·96	0·3	1·14	0·3	<0·001
Body fat (%)	34·0	6·8	37·1	5·8	32·5	6·3	34·1	6·4	34·5	6·6	<0·001
Fat to muscle ratio	1·00	0·3	1·13	0·2	0·95	0·2	1·02	0·2	1·02	0·2	0·001
Total cholesterol (mg/dl)	191·5	32·3	186·6	33·9	206·3	40·9	188·4	34·2	191·9	34·6	0·007
HDL-cholesterol (mg/dl)	60·3	14·8	57·4	12·6	66·3	15·9	59·9	14·3	60·4	14·6	0·006
FRS (%)	5·82	4·53	7·55	4·21	5·25	3·70	7·74	6·06	6·46	4·77	0·001
	*n*	%	*n*	%	*n*	%	*n*	%	*n*	%	
Categorical variables†											
Smoking	2	1·0	0	0	1	1·8	2	2·7	5	1·2	0·349
Alcohol drinking	11	5·7	1	1·1	1	1·8	0	0	13	3·2	0·071
Hypertension	63	32·9	40	47·1	12	22·2	36	48·6	151	37·3	0·002
Diabetes mellitus	26	13·6	16	18·8	5	9·2	12	16·2	59	14·6	0·417
Stroke	1	0·5	1	1·1	0	0	4	5·4	6	1·4	0·047
Metabolic syndrome	54	28·2	29	34·1	5	9·2	18	24·3	106	26·2	0·005

BP, blood pressure; BMI, body mass index; HDL, high-density lipoprotein; SMI, skeletal muscle mass index; FRS, Framingham risk scores.

* Values in the continuous variables were expressed as mean and standard deviation unless otherwise specified.

† Values in the categorical variables were expressed as number (percent).

### Association between different sarcopenia degrees and cardiovascular risk

Table 3 displayed the association between different sarcopenia degrees and the odds ratio (OR) of higher FRS in men and women. After adjustment of pertinent variables, men with dynapenia had significantly higher FRS than presarcopenia group with OR of 2·52 (95 % confidence interval (CI): 1·03, 6·14). However, no significant finding was found in participants with sarcopenia. In terms of women, those with sarcopenia had significantly higher FRS than presarcopenia group with OR of 2·81 (95 % CI: 1·09, 7·27) after adjusting variables.

**Table 3:** Comparison of Framingham risk scores among four groups of participants in both genders

		Robust			Dynapenia			Presarcopenia	Sarcopenia		
Gender	Models*	OR	95 % CI	*P*	OR	95 % CI	*P*		OR	95% CI	*P*
Male	unadjusted	2·09	1·01, 4·36	0·048	2·52	1·04, 6·09	0·040	1	1·15	0·43, 3·09	0·773
	adjusted	2·21	1·06, 4·65	0·035	2·52	1·03, 6·14	0·043	1	1·12	0·42, 3·02	0·818
Female	unadjusted	1·61	0·67, 3·85	0·282	2·79	1·11, 7·02	0·029	1	2·84	1·11, 7·25	0·029
	adjusted	1·58	0·66, 3·79	0·304	2·62	1·03, 6·64	0·041	1	2·81	1·09, 7·27	0·033

CI, confidence interval; OR, odds ratio.

* Adjusted covariates: alcohol consumption and history of stroke.

## Discussion

Several researchers propose that the prevalence of sarcopenia is related to CVD risks in elderly adults. Numerous evidence have reported that patients with low handgrip strength are at risk of CVD mortality^(22)^. In a cross-sectional study, physical disability is suggested an important risk factor for CVD mortality in elderly adults^(23)^. Recently, higher levels of skeletal muscle mass was proposed to be linked to lower 10-year CVD risk^(24)^. In comparison with previous studies, we categorised participants into dynapenia, presarcopenia and sarcopenia groups based on the criteria rather than individual components. We demonstrated a gender difference that older dynapenic men and older sarcopenic women had higher risks of 10-year CHD, respectively. To the best of our knowledge, our study was the first to compare the relationships between different sarcopenia degrees and CVD risk in an elderly population.

Sarcopenia is considered as a precipitating factor of insulin resistance and glucose metabolism in an elderly population^(25)^. Sarcopenic obesity is also suggested to have a positive association with metabolic syndrome in adults^(26,27)^. Kim et al. demonstrated that sarcopenic obesity was significantly associated with increased CVD risks such as glucose intolerance and metabolic syndrome in Korean adults^(17)^. Smoking was considered an important risk factor of sarcopenia, especially in men^(28)^. The different anthropometric index also had gender difference in the effect on CVD risk^(29)^. Accordingly, loss of skeletal muscle mass is indicated to have a relation with several health outcomes. In a cross-sectional study that included 3042 adults, Tyrovolas et al. reported that decline of skeletal muscle mass was associated with an increased 10-year cardiovascular incidence in those older than 45 years of age^(24)^. The muscle cells exert an endocrine function by secreting myokines with beneficial effects on the cardiovascular system^(30)^. Loss of skeletal muscle mass and impaired endocrine function could lead to poor CVD outcomes^(31)^; however, the detailed mechanisms should be further investigated. In addition, skeletal muscle mass is known for its major role in the glucose metabolism pathway in the human body^(32)^. Impaired glucose metabolism and altered insulin resistance caused by decreased skeletal muscle mass have an adverse impact on CVD events^(33,34)^.

Epidemiological data for the prevalence of sarcopenia and dynapenia between older men and women are conflicting^(35,36)^. Several studies demonstrate that men have greater muscle loss than women in older population^(37)^. Fried et al. proposed that women were more likely to develop frailty and sarcopenia because women had lower muscle mass and muscle strength than men at the same age^(38)^. In the present study, we demonstrated that older women with sarcopenia had a higher CVD risk than relative healthy participants.

Testosterone levels are suggested to be associated with decreased muscle quality^(39)^. Testosterone supplementation stimulates muscle protein synthesis and inhibits muscle protein degradation leading to the contribution of muscle fibre hypertrophy^(40)^. Low testosterone level is considered an indicator of sarcopenia and may lead to the development of frailty in older men^(41)^. In a population-based study that included 927 Taiwanese, Lee et al. reported that handgrip strength was related to FRS in men but not in women^(42)^. It is consistent with our finding that male participants with dynapenia have significantly higher FRS than females. However, the underlying mechanisms for relationships between decline of muscle mass, muscle strength and risk of CVD have not been clearly recognised, and it deserved to be explored in future studies.

The strength of this study deserved to be addressed. Previous researches defined muscle mass and muscle function by variety of indicators. We defined the cut-off point of low muscle mass, low muscle strength and low physical performance according to the consensus of AWGS. Instead of estimating the correlation between single muscle health indicator and cardiovascular outcome, we categorised these muscle parameters into dynapenia, presarcopenia and sarcopenia to adapt the current critical issue of geriatric syndrome in clinical practice. From this study, it is given a hint for medical providers to take caution on changes of muscle quantity and quality among old people in clinical practice. In addition, public health interventions are required for developing effective strategies to promote prevention and treatment of low muscle mass and low muscle function in general practice.

There are some limitations that should be noted in the present study. The cross-sectional study design limited us to identify the causal relationships between different sarcopenia degrees and 10-year CVD risk. A long-term observation period should be considered in future studies. Second, we included Taiwanese participants in this study. Extrapolation of our findings to other ethnicities may be limited. Third, the CVD risk estimated by the FRS was not an actual CVD outcome. The Framingham risk model was originally applied for Western populations. Lastly, it may not reflect the different genetic, environmental and social factors in the Taiwanese population.

## Conclusion

In summary, we found that elderly men with dynapenia and elderly women with sarcopenia were independently associated with increased FRS scores. From this study, we recognise that old people with low muscle mass or low muscle function have an increased risk of CVD. Further studies on the causal effect of sarcopenia on CVD risk were required, which might not only expand understanding of the mechanism in our study but also establish new prevention strategies of muscle quantity and quality for old people in clinical practice.
